# Salivary Total Antioxidant Capacity and Lipid Peroxidation in Patients with Erosive Oral Lichen Planus

**DOI:** 10.5681/joddd.2014.006

**Published:** 2014-03-05

**Authors:** Atena Shirzad, Mahdi Pouramir, Maryam Seyedmajidi, Niloofar Jenabian, Ali Bijani, Mina Motallebnejad

**Affiliations:** ^1^Assistant Professor, Department of Oral Medicine, Dental School, Babol University of Medical Sciences, Babol, Iran; ^2^Associate Professor, Department of Biochemistry, Babol University of Medical Sciences, Babol, Iran; ^3^Assistant Professor, Dental Materials Research Center, Dental School, Babol University of Medical Sciences, Babol, Iran; ^4^Assistant Professor, Department of Periodontology, Dental School, Babol University of Medical Sciences, Babol, Iran; ^5^MD, Babol University of Medical Sciences, Babol, Iran; ^6^Associate Professor, Cellular and Molecular Biology Research Center, Dental School, Babol University of Medical Sciences, Babol, Iran

**Keywords:** Antioxidant, lipid peroxidation, oral lichen planus, saliva

## Abstract

***Background and aims.*** Oral lichen planus is a common chronic inflammatory disease of the oral mucosa with malignant potential, pathogenesis of which is not still well known. Free radicals and reactive oxygen species can play an important role in the pathogenesis of oral lichen planus. The aim of this study was to investigate salivary oxidative stress and antioxidant systems in patients with oral lichen planus.

***Materials and methods.***In this case-control study, 30 patients with oral lichen planus (case group) and 30 age-and gender-matched healthy subjects (control group), referring to Dental School of Babol University of Medical Sciences, were selected using simple sampling method. Unstimulated saliva of the two groups was collected. Salivary total antioxidant capacity (TAC) and lipid peroxidation products were investigated and compared, using ferric reducing antioxidant power (FRAP) and thiobarbituric acid reactive substance (TBARS) methods, respectively. Data were analyzed using Student' t-test.

***Results.*** The mean and standard deviation of salivary TAC in patients with oral lichen planus (297.23 ± 149.72 μM) was significantly lower than that in the controls (791.43 ± 183.95 μM; P & 0.0001), and mean and standard deviation of salivary malondialdehyde (MDA) (0.49 ± 0.30 μM) was remarkably higher in oral lichen planus patients compared to the control group (0.15 ± 0.11 μM) (P & 0.0001). TAC was also reduced in both groups in line with an increase in the level of MDA (P & 0.0001, r = -0.48).

***Conclusion.*** The results of this study suggested that an increase in oxidative stress and an imbalance in antioxidant defense system in the saliva of oral lichen planus patients may be involved in the pathogenesis of oral lichen planus.

## Introduction

Oral lichen planus (OLP) is a common chronic inflammatory disease of the oral mucosa with malignant potential.^1^ The clinical presentation of OLP has several forms (reticular, popular, erosive/ulcerative, bullous, plaque-like) that can cause symptoms ranging from a burning sensation to severe pain interfering with speaking, eating and swallowing.^1^ However, the exact pathogenesis of the disease is still unknown.^2^ Several studies have pointed to the important role of oxidative stress in the pathogenesis of autoimmune and inflammatory skin diseases; for instance, psoriasis and other T-cell-mediated skin disorders are indicative of increased oxidative stress and decreased antioxidant capacity.^3^ In recent years, many studies have suggested the role of oxidative stress in the etiology of oral lichen planus as an autoimmune disease.^4-8^ There are various antioxidant systems in body fluids. On the other hand, antioxidant evaluation methods are numerous. Recent studies on oral lichen planus have used different methods and assessed antioxidant components.^4,7,8^Total antioxidant capacity (TAC) shows a combination of all antioxidant activity. Therefore, it can be a good marker for evaluation; as a result, we used this marker in this study. Although lipid, proteins and DNA molecules may be affected during oxidative stress,^3^ we only used lipid peroxidation as a marker of oxidative stress in our study. Other studies have evaluated serum oxidative systems but we used saliva instead.^4,7^

Oxidative stress which is defined as a disruption in the balance between oxidants and antioxidants leads to overproduction of free radicals and reactive oxygen species (ROS); the pathology associated with ROS is derived from their ability to modify cellular and extracellular macromolecules, such as proteins, lipids, and DNA to disrupt cellular function. Through oxidation of unsaturated fatty acids in cell membranes or lipoproteins, ROS induces lipid peroxidation which appears to be a major manifestation of oxidative stress.^9^Research has demonstrated that increased lipid peroxidation in cell membrane can stimulate inflammatory and immune responses.^10^Malondialdehyde (MDA) as the most important product of lipid peroxidation can be used as a marker for the measurement of oxidative stress.^11^Oxidative stress can also be evaluated by the measurement of antioxidant capacity in biological fluids.^12^ Since the body antioxidants are highly varied, use of an index that measures total capacity of antioxidants in biological fluids, such as TAC, could be a new and suitable method.^13^ Like other biological fluids, saliva as the body's first line of defense in the oral cavity, confronts with oxidative stress-induced free radicals through a variety of mechanisms such as enzymatic and non-enzymatic defense systems.^14^ In this regard, the present study aimed to investigate oxidative stress condition and antioxidant defense system in patients with oral lichen planus and to compare it with that of healthy individuals through measuring MDA and TAC levels of non-stimulated saliva.

## Materials and Methods

This case-control study was performed on a sample of patients who were admitted to the Department of Oral Medicine, Faculty of Dentistry, Babol University of Medical Sciences, Iran, using the simple sampling method in 2010-2011. This study was approved by the Ethics Committee of Babol University of Medical Sciences (ORN: 1040). Patients also gave an informed consent for saliva sampling.

The sample size was determined according to previous studies.^4,6^In this study, the case group included 30 patients with OLP, ranging in age from 25 to 46 years, whose disease was confirmed by clinical (the presence of bilateral lesions or reticular lesions in the oral cavity) and histopathological examination. In the control group, 30 healthy individuals with no lichen planus were selected, with an age range of 24-48 years, matched with the case group in terms of age and gender.

Inclusion criteria for both groups consisted of receiving no immunosuppressive, non-steroidal anti-inflammatory and systemic steroids, as well as antioxidants and vitamin supplements since four weeks before the operation, or local steroids drugs two weeks before surgery, no history of trauma and surgery four weeks before saliva sampling, no disease affecting the immune system such as AIDS, rheumatoid arthritis and other inflammatory disorders, no history of malignancy, no periodontal disease (examined by a periodontist), no smoking, and no active dental decay. Information associated with the case and the control groups was written in the relevant forms.

For collecting unstimulated saliva samples, all the participants were asked to avoid eating, drinking and toothbrushing 90 minutes before sampling. All the samples were collected by spitting in the morning from 9 to 11 a.m.^15^ Following collecting saliva samples in the test tubes, the tube caps were tightly closed; the samples were immediately transported to the biochemistry laboratory after being encoded regarding the study groups.

It is worth mentioning that all the chemical materials of the study, except TPTZ (Sigma- Aldrich, Germany), were obtained from Merck Company (Darmstadt, Germany). Saliva samples were centrifuged, (Clement 2000, North Sydney, Australia) at 2000 rpm for 10 min, so that the debris could be separated. The supernatant was then transferred to microtubes, encoded and kept at -80°C until the time of experiment. Salivary total antioxidant capacity was measured by FRAP technique^16^ as salivary antioxidants reduce Fe^3+^, present in FRAP reagent, and a color is produced as a result. FRAP working solution consists of 10 mM TPTZ (2,4,6-tripyridyl-s-triazine) in 40 mM HCL plus FeCl_3 _and 0.3 M buffer acetate (pH=3.6) in the ratio of 10:1:1.

1.5 mL of FRAP working solution was brought to 37°C, and 50 μL of saliva was then added to the solution so that the reaction could be initiated. The absorption of samples and standard solution, containing different concentrations of FeSO_4_.7H_2_O (125, 250, 500, 1000 µM), were read at 593 nm using a spectrophotometer (Jenway, Statfordshire, UK). Salivary antioxidant concentration was obtained by comparing the optical absorbance of samples with that of standard solution in the standard curve.

MDA, as a product of lipid peroxidation, was measured through the TBARS technique,^17^in which MDA reacts with TBA (thiobarbituric acid) at 90-100°C, pH=2-3, within 15 minutes to produce a pink pigment. One volume of the sample was mixed with two volumes of the solution containing 15% trichloroacetic acid (w/v), 0.375% TBA (w/v) and 0.25 N hydrochloric acid, and the mixture was placed in boiling water bath for 30 minutes. After cooling, the samples were centrifuged again, at 3000 rpm for 15 min, and the absorption was read at 532 nm by the spectrophotometer. TBARS was finally calculated with respect to the molar absorption coefficient of 1.56×10^5^M^-1^cm^-1^.

Data were analyzed by SPSS 16 statistical software and were presented as means and standard deviations. Student’s t-test was used for independent variables, and correlation assessments were performed by using Pearson´s correlation analysis. P < 0.05 was considered statistically significant.

## Results

**Figure 1.**
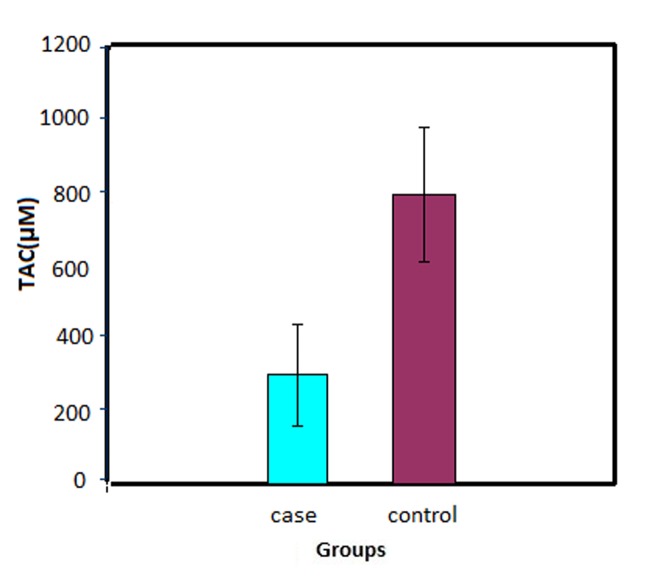
The unstimulated whole saliva concentration of total antioxidant capacity (TAC) in the case (patients with oral lichen planus) and control (healthy individuals) groups.

**Figure 2.**
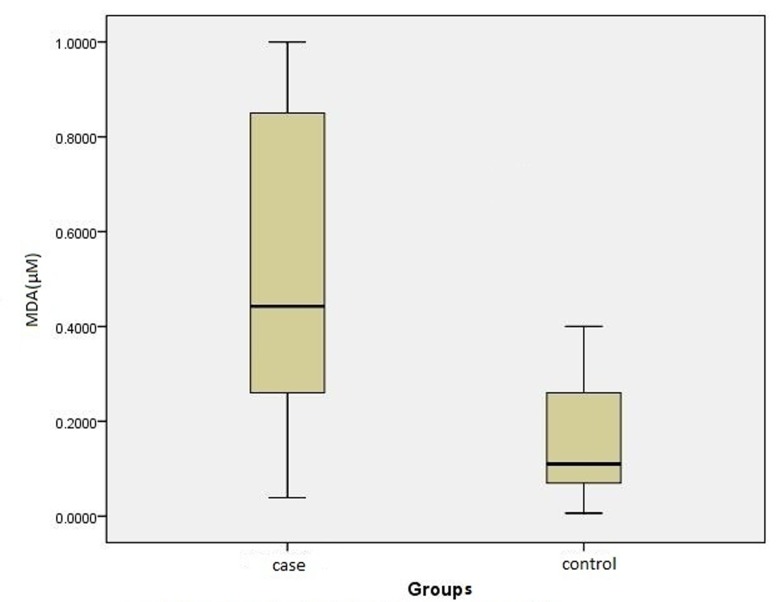
The unstimulated whole saliva concentration of malondialdehyde (MDA) in the case (patients with oral lichen planus) and control (healthy individuals) groups.

A total of 26 females and 4 males with a mean age of 44.37 ± 8.16 and 44.77 ± 8.61 years were included in the case and the control groups, respectively; 29 patients had erosive lichen planus and one had plaque-like lichen planus. The mean TAC in the case group (297.23 ± 149.72 μM) was significantly lower than that in the control group (791.43 ± 183.95 μM) (P < 0.0001) (Figure 1); the mean MDA was remarkably higher in the case (0.49 ± 0.30 μM) than in the control group (0.15 ± 0.11 μM; P < 0.0001; Figure 2).

There was an inverse relationship between TAC and MDA (P < 0.0001), i.e. a decrease in MDA level was accompanied by an increase in TAC.

No significant relationship was observed in MDA and TAC levels with age (P = 0.51, P = 0.91) and disease duration (P = 0.81, P = 0.38) in the case group.

## Discussion

In the present study, MDA and TAC levels of unstimulated saliva in patients with oral lichen planus were compared with those of healthy individuals and it was shown that MDA level was significantly higher in the saliva of OLP patients compared to the control group and salivary TAC was remarkably higher in OLP patients compared to the control group.

Ergun et al^8^ found that salivary and plasma levels of MDA were significantly higher in OLP patients compared to healthy subjects. In an investigation by Sander et al on the serum of OLP and healthy participants, an increase in MDA as well as AoHdG (as a sign of DNA damage) was observed in OLP patients.^3^ Recent findings indicate that an increase in oxidant levels occurs along with an increased damage to lipids, DNA and serum proteins in OLP subjects, which is in line with the present research.^3^

The present study also showed lower levels of TAC in OLP patients compared to the controls, indicating decreased antioxidant activity due to oxidative stress.The serum TAA in OLP patients was lower than the healthy individuals in the study of Ergun et al, which is consistent with the present research, since TAA is also a sign of total antioxidant capacity but has been measured by other biochemical methods.^8^ On the other hand, TAC level was higher in OLP patients compared to the controls, consistent with the study of Agha-Hosseini et al.^6^ They suggested that such a discrepancy can be attributed to two possible reasons in the process of oxidative stress; first, the increase in ROS is observed with or without an increase in antioxidant system; second, antioxidant reduction occurs without any significant alteration in ROS.^6^

Evaluation of antioxidant enzymes, CAT and SOD by Sezer et al^4^ revealed an increased level of CAT and a decreased level of SOD, and the reason behind this antioxidant imbalance was attributed by the researcher to increased H_2_O_2_. Therefore, this study is also consistent with the present investigation in terms of impairment in antioxidant balance in the process of oxidative stress.

Similar to the findings achieved by Ergun et al,^8^ there was an inverse relationship between the level of unstimulated salivary MDA and TAC in OLP and healthy subjects in this study; this implies reduced antioxidant intake with increased lipid peroxidation in the process of oxidative stress which, per se, shows lack of balance between production of free radicals and antioxidants.

There is no balance between free radical production and level of antioxidant activity in the process of oxidative stress, leading to elevated ROS levels in the biological environment. ROS overproduction results in damage to the body biomolecules such as DNA, amino acids, carbohydrates and lipids and eventually disturbance of structural organization and cell function.

Measuring the oxidation products is the most direct method for the evaluation of oxidative stress. Antioxidant system of the body is very diverse and complex and includes a variety of molecules and enzymes inside and outside the cell body in all the biological fluids such as plasma and saliva.^18-20^

Since free radical-scavenging antioxidants are consumed at a higher rate with enhanced ROS activity during the process of oxidative stress, activity of antioxidant components can indirectly examine the activity of oxidative stress.^21,22^ Although investigating an antioxidant alone may not be indicative of the antioxidant activity of all the biological fluids, evaluation of several different antioxidants is difficult, time-consuming and expensive, and mutual effects of antioxidants are also added. To this end, use of the TAC index that represents the cumulative effect of all the antioxidants in biological fluids is indicated.^12,23^

Several studies have revealed effective use of antioxidants in controlling the activity of oxidative stress-associated disorders such as vitiligo, Behçet's disease and lichen sclerosus.^24-26^ Rai et al^27^ also showed improvement in OLP due to curcumin antioxidant property. These therapeutic effects indirectly underline the role of oxidative stress in the pathogenesis of OLP.

There is some data that pathophisiology of lichen planus lesions is related to inflammatory processes which activate inflammatory cells to release ROS due to the phenomenon of respiratory burst. This phenomenon damages adjacent cells.^28^There is also a hypothesis that lichen planus is a delayed type of hypersensitivity response that activates T cells to release inflammatory cytokines. These cytokines stimulate keratinocytes to increase ROS production.^29^ Accordingly, a study by Sander showed that reduced antioxidant capacity in OLP may result in increased oxidative process-mediated cellular damage.^3^ The results of the present study are consistent with the above findings in terms of lack of balance between antioxidants and ROS in OLP. Therefore, it is recommended that further studies be conducted on the therapeutic effects of antioxidant-containing medications to develop newer and better treatment for OLP. One of the problems in this study was investigation of only erosive type of OLP; further studies are suggested with larger sample size to resolve this problem.

## Conclusion

The present study demonstrated an imbalance between salivary total antioxidant capacity and increased oxidant levels in patients with OLP.

##  Acknowledgments

Hereby, the authors would like to thank Deputy of Research and Technology of Babol University of Medical Sciences for financial support of the project.
